# Characterisation of strength and deformation characteristics of alkali-activated rice husk ash filler-assemblage perimeter rock

**DOI:** 10.1038/s41598-023-43870-9

**Published:** 2023-10-02

**Authors:** Wenhua Zhao, Ruilin He, Qi Sun, Qi Gu

**Affiliations:** 1 College of Architecture and Transportation, Liaoning Technical University, Fuxin, 123000 China; 2 School of Civil Engineering, Liaoning Technical University, Fuxin, 123000 China

**Keywords:** Engineering, Materials science

## Abstract

In this study, the strength characteristics, deformation characteristics and damage characteristics of three kinds of specimens, namely, surrounding rock, cemented paste backfill (CPB) and a surrounding rock-CPB combination, were studied by uniaxial compression testing using rice husk ash and slag as cementing materials, and the mechanical properties of the combination specimens with different height ratios were also analyzed. The results showed that the surrounding rock specimens were the strongest, followed by the composite body, and the CPB was the weakest. The relationship between different height ratios of the assemblage and the cut line modulus was found according to the fitted curves. The CPB specimens and the surrounding rock specimens showed ductile damage, while the assemblage specimens showed brittle damage.

## Introduction

Mineral resources are the essential material basis of China's economy, and mining results in the formation of a many areas where rock was removed, resulting in surface subsidence, subsidence and deformation and other destructive phenomena^1–3^. There are many hidden dangers in mines, and safety is a major cause of concern. To solve the safety problems of mining areas after mining operations have ceased, paste filling methods are widely used to solve the problems of uneven settlement and collapse in areas where rock was removed^4,5^. Cement, the cementitious material that was used in previous fillings, not only consumes a large amount of energy but also causes some environmental impact. The search for a replacement for cement has become a direction of development for filling technology^6^. The use of solid waste as the main research material for CPB can make full use of resources and at the same time protect the environment. Thus, finding a CPB material that can meet production needs and is economical and reasonable will be the key to solving the problem^7,8^.

RHA is a waste residue from the combustion of rice husk fuel, and its main component is SiO_2_, which can be used as a CPB cementitious material^9,10^. Scanning electron microscopy (SEM) was used to observe the microstructure, and the observations showed that the C–S–H gel produced by the hydration of RHA was dense and acted as a CPB^11^. RHA replacement of cement improved the compressive, tensile and flexural strength of concrete^12,13^, which reduced the creep of concrete and improved the durability and uniformity of concrete^14,15^. Similar to the current common mineral admixtures, such as silica fume (SF)^16^, slag (SS)^17^, and fly ash (FA)^18^, RHA has high volcanic ash activity and is also added to concrete.

In underground mineral mining, CPB is used in the mining void to support the rock around the quarry by forming a common load-bearing CPB-perimeter rock laminar combination, which is crucial to mine stability. The mechanical properties of the CPB materials and the interaction mechanism and combination form have a significant effect on the overall synergistic deformation mechanism and the support effect of the surrounding rock. In view of the importance of the CPB-perimeter rock laminate assemblage, scholars have explored the mechanical properties and proportional optimisation design of the assemblage in recent years. Wang et al.^19^ investigated the compressive mechanism of rock package fillers and found that encapsulated rock could enhance the uniaxial compressive strength (UCS) and elastic modulus (EM) of the backfill material. Fu et al.^20^ investigated the effect of structural parameters on the creep mechanical properties of composite specimens of surrounding rock and filling body (SR-FB). Li et al.^21^ conducted uniaxial compression tests on coal-rock, rock-coal and rock-coal-rock combination samples. It was concluded that the rock-coal-rock combination samples exhibited plastic damage, while the coal-rock and rock-coal combination samples exhibited brittle damage. Zhao et al.^22^ investigated the synergistic deformation mechanism of CPB specimens consisting of tantalum-niobium mine tailings and rocks under different ratios of cement tailings, and the results showed that the overall strength of the composite was comparable to the strength of a single CPB specimen, the modulus of elasticity increased with the decrease in the strength ratio of the specimen, and the damage modes of the CPB mainly exhibited shear damage and tensile damage. He et al.^23^ performed uniaxial compression acoustic emission (AE) experiments on single cement stone and cement stone-rock combinations with different cement tailings ratios to study the crack evolution of samples at different stages. Liu et al.^24^ investigated the mechanical properties of coal in coal-rock assemblages, and the compressive strength of coal-rock specimens increased with increasing rock strength and decreased with increasing coal-rock height ratio. Pan et al.^25^ investigated the deformation characteristics and loading damage of coal-rock assemblages with different coal-rock height ratios and found that the uniaxial compressive strength of coal-rock assemblages increased with increasing coal-rock height. Chen et al.^26^ conducted experimental and numerical studies on the response of rock-coal, coal-rock and rock-coal-rock composite structures under triaxial compression. The experimental data showed that the mechanical parameters of rock-coal-rock composites were better than those of rock-coal and coal-rock combinations, and the failure of rock assemblages mainly occurred in coal. Yu et al.^27^ conducted triaxial compression tests on laboratory specimens containing rock and backfill to understand the effect of the rock/backfill volume fraction and perimeter pressure on strength and fracturing in rock backfill systems. Li et al.^28^ conducted uniaxial compression tests on composite samples consisting of sandstone and cemented gangue-fly ash backfill (CGFB) to investigate their deformation and damage characteristics. Tan et al.^29^ designed a composite structure made of cement tailings backfill (CTB) and rock core (RC) and investigated its mechanical properties, damage process, damage characteristics and microstructural features. Liang et al.^30^ analyzed the displacement and stress field evolution characteristics of the overlying strata in backfill mining by physical modeling, and the backfill improved the stress distribution, reduced the stress concentration in the surrounding rock, and helped to prevent the progressive destruction of the overlying strata. Li et al.^31^ investigated the creep properties of rock bodies under different seepage conditions and effectively described the whole change process of rock creep properties according to the model.

Most of the above studies focused on the mechanical properties of different composite specimens under uniaxial conditions, and there were fewer studies on different heights and types of CPB-perimeter rock composites and fewer studies on RHA slag as a cementing material. In this study, five different types of CPB-surrounding rock combinations with height ratios of 1:9, 1:3, 3:1, 9:1, and 1:1 and two different types of CPB-surrounding rock combinations were used to compare and analyze their cut-line modulus values and compressive strength fitting curves. The stress‒strain curves and deformation damage characteristics between the combinations and single CPB and single surrounding rock were compared and analyzed to provide a basis for evaluating destabilization damage in mines. The results of this study have some significance to guide the practices of mining and filling voids in mines.

## Experiment

### Raw materials

The main materials for this experiment consisted of RHA, slag, aggregate, water glass and NaOH. The RHA was taken from a mining area in Hubei, and impurities were removed through simple screening. The slag was made from S95 slag produced by Shandong Kangjing New Material Technology Co., Ltd. with the main chemical composition of CaO, SiO_2_ and Al_2_O_3_, and the aggregate was made from tailing sand taken from Fuxin Mongol Autonomous County Yuan Phosphorus Mining Co. The alkali exciter was water–glass from Jiashan Yourui Refractories, and NaOH was used to adjust it to the modulus required for the experiments.

### Test conditions

**Figure 1:**
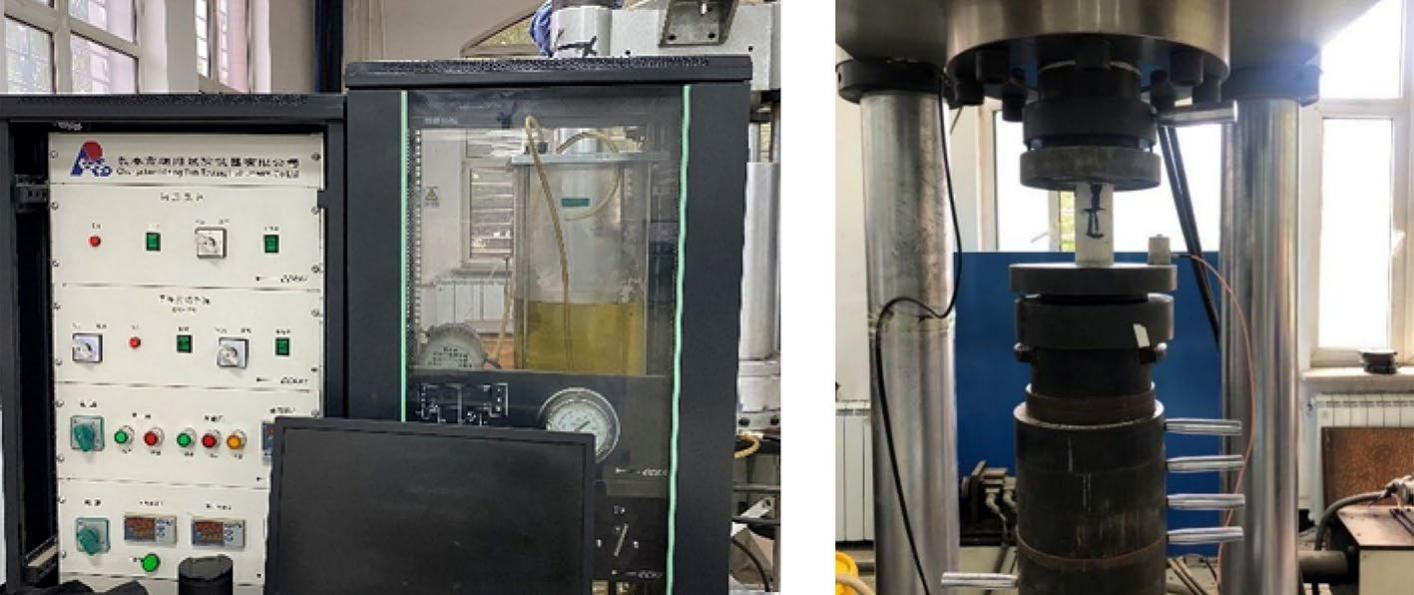
Rock triaxial testing machine.

The uniaxial compressive strength of the rock was tested using a triaxial testing machine (model TAW-2000), as shown in Fig. 1. The mold loading method was displacement-controlled, the specimen loading rate was 1 mm/min, and the displacement limit was 5 mm. An NJ-160A cement net slurry mixer was used to mix the net slurry material and prepare the slurry. A YH-90B constant temperature and humidity curing box was used to maintain the net slurry specimens, the CPB specimens and the combined specimens.

### Test preparation

The gelling materials used in this experiment were RHA and slag, the aggregate was tailing sand, and the exciter solution materials were NaOH, water glass and water. The RHA accounted for 10% of the cementing material, the fixed slurry concentration was 83%, the alkali-activated modulus was 1.0, the amount of alkali used was 4% of the mass of the cementing material, and the water-cement ratio was 0.35^32^. The above materials and proportions were used to make the surrounding rock slurry and filling slurry, and the slurry was poured into the Φ 50 mm × L 100 mm mold that had been well oiled. After 1 d, the slurry was shaped and demolded and then placed in a constant temperature and humidity curing chamber (20 ± 1 °C, humidity > 95%) for 28 d to obtain the specimens of the surrounding rock and CPB. The experiments were conducted in strict accordance with the GB/T50266-2013 Engineering Rock Test Standard, and the uniaxial compressive strength was measured using a rock triaxial testing machine. The experimental results are shown in Table 1.

**Table 1:** Strength of the CPB and surrounding rock.

Type	Number	Uniaxial compressive strength/MPa	Height/mm	Diameter/mm
Single CPB Specimen (3 pcs.)	T1	7.06	101.02	50.12
	T2	7.07	102.35	50.27
	T3	7.27	102.13	50.42
Average value	T	7.13	101.84	50.27
Surrounding rock specimen (3 pcs.)	J1	30.27	99.97	50.32
	J2	32.58	100.42	50.55
	J3	31.21	99.84	50.03
Average value	J	31.35	100.08	50.30

**Figure 2:**
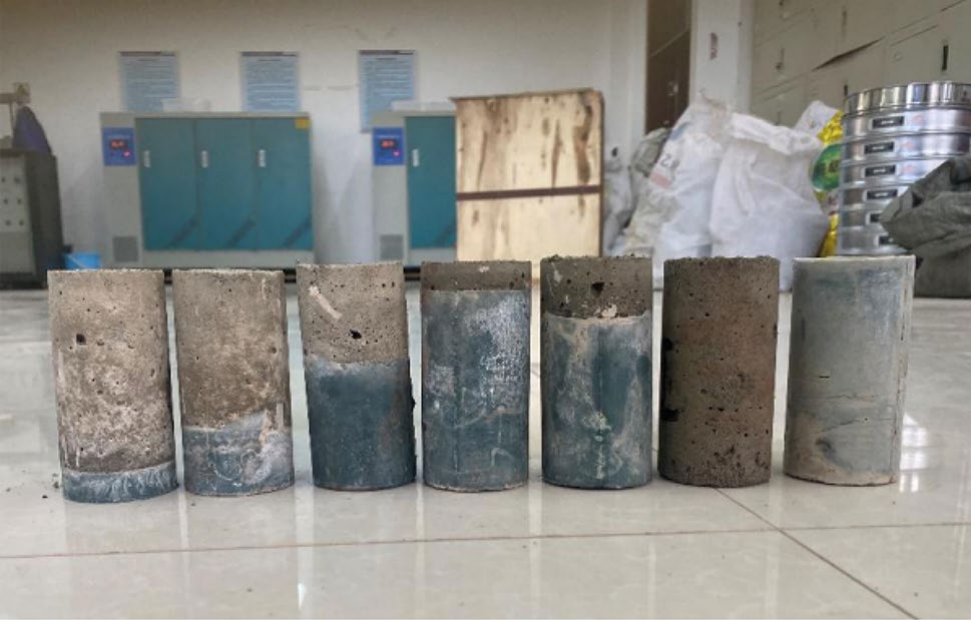
Assemblies at each heights.

The test combination as shown in Fig. 2 was divided into two parts, upper and lower, using net slurry to simulate the strength of the surrounding rock. The specimens were set in two different combinations as shown in Table 2: 1 ~ 3 were the specimens of the surrounding rock under the upper CPB, and 4 ~ 6 were the specimens of the surrounding rock under the CPB. According to the different heights of the net slurry and CPB settings, five different height ratio combinations were designed. The height ratio refers to the different thicknesses of the net slurry and CPB in the combination specimens, which were 1:9, 1:3, 3:1, 9:1, and 1:1 and marked as A, B, C, D, and E, respectively. Each group contained three of the same type of specimens. When the specimens were of type A, each group was marked as A1, A2, and A3 when the upper layer of CPB was under the surrounding rock. When the upper layer of surrounding rock was under the CPB, each group was marked as A4, A5, and A6, respectively, and there were three specimens of each type. The assemblage was poured in layers according to different thickness ratios, and the slurry was poured into a Φ 50 mm × L 100 mm mold that had been brushed with oil.

**Table 2:** Assembly test sheet.

Type	Number		Height ratio	Number of specimens
Upper CPB and lower surrounding rock	A	A1 ~ A3	1:9	3
	B	B1 ~ B3	1:3	3
	C	C1 ~ C3	3:1	3
	D	D1 ~ D3	9:1	3
	E	E1 ~ E3	1:1	3
Upper surrounding rock and lower CPB	A	A4 ~ A6	1:9	3
	B	B4 ~ B6	1:3	3
	C	C4 ~ C6	3:1	3
	D	D4 ~ D6	9:1	3
	E	E4 ~ E6	1:1	3

## Mechanical properties

The unconfined compressive strength test was performed on three groups of specimens of CPB, net slurry and combined body and the surfaces of the specimens were polished smooth before testing. The corresponding mechanical test data were obtained by using a TAW-2000 rock triaxial testing machine. There were three CPB specimens, T1 ~ T3, and three net slurry specimens, J1 ~ J3, in each group, and there were six CPB specimens, A1 ~ A6, B1 ~ B6, C1 ~ C6, D1 ~ D6, and E1 ~ E6 in each group, totaling 36 specimens. As the composition and structure of the materials of each specimen were different, the magnitudes of their strengths differed greatly, and the corresponding changes in each type of specimen were studied by stress‒strain curves.

### Strength characteristics of CPB specimens

**Figure 3:**
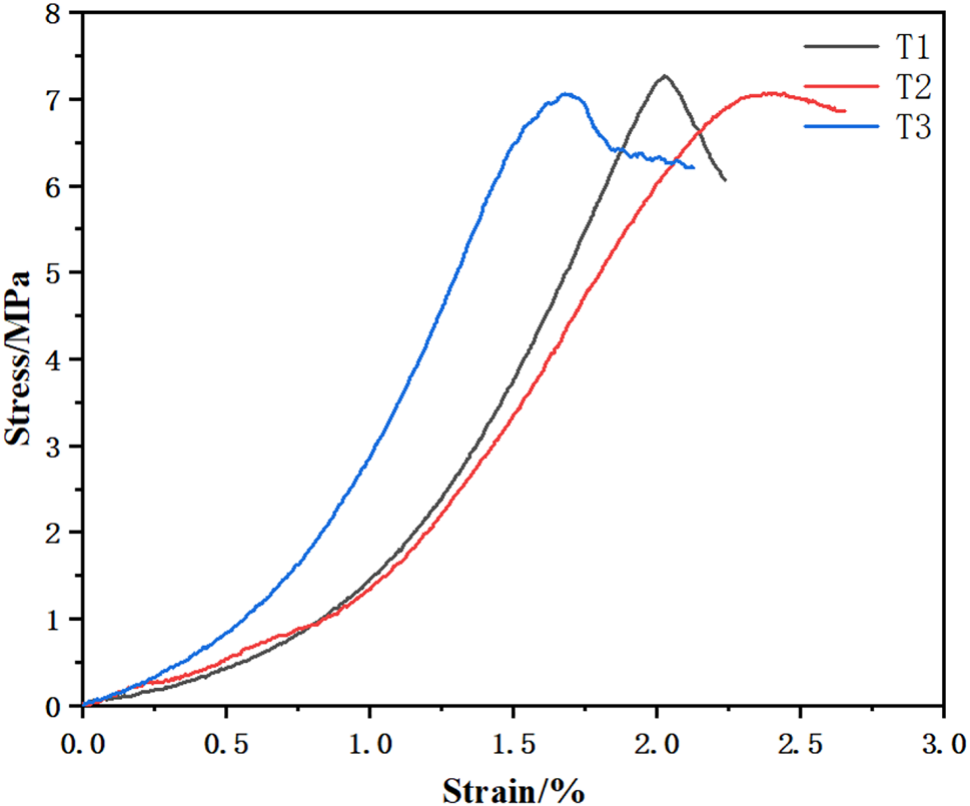
Curve of the CPB.

The stress‒strain diagram of the T group CPB specimen is shown in Fig. 3 below. When the stress rose to 3 MPa, the strain was between 0 and 1.0. At this time, the specimen was in the dense stage, the pores were compacted under the action of external force, there was rising trend of the curve, and the line was concave downward. When the stress increased to 3–6.5 MPa, the specimens was in the elastic stage, the curve was close to a straight line, and the stress‒strain relationship was proportional. Then, the curve continued to rise to the peak stress, and was convex upward to the peak when the growth rate slowed, showing yield stress. After the peak stress, the stress decreased, the T3 curve declined slowly, and the specimen maintained a certain residual stress.

### Strength characteristics of surrounding rock specimens

**Figure 4:**
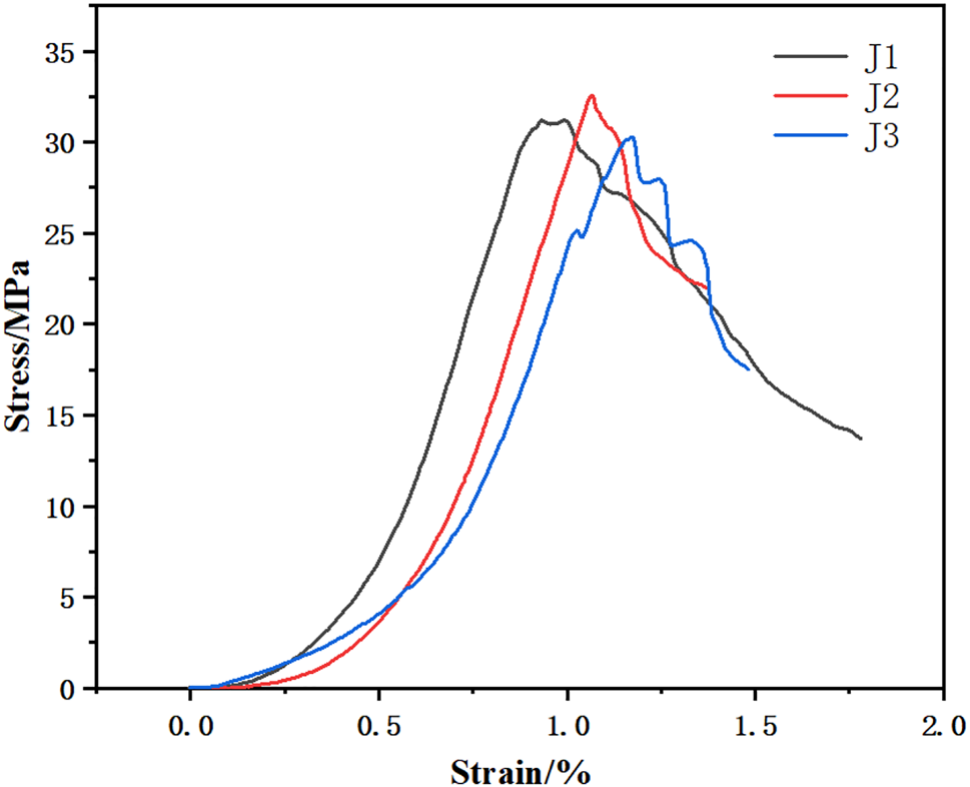
Surrounding rock curves.

The changing patterns of the stress‒strain curves of three net slurry specimens at an age of 28 d under uniaxial compression testing are shown in Fig. 4 below. The specimens were tested for their resistance to damage and deformation by compression under the action of external forces, and the specimens underwent strain during the compression process. The mechanical characteristics of the specimens and the trends of change were reflected by the relationship between the stress‒strain curve, which showed an increasing trend and then a decreasing trend, and the curve was divided into two parts, i.e., rising and falling sections. The specimens were subjected to external loading. Small cracks developed first in the net slurry specimens and expanded to penetration damage. Before the peak, the stress‒strain curve grew steeply, and after reaching the peak, the stress‒strain curve started to decline.

As shown in the figure, the specimens of Group J were in contact with the top plate of the press when the strain was 0 ~ 0.2. In the rising stage, the stress increased to 10 MPa, the strain was 0.2 ~ 0.7, and the curve was depressed and bent downward. When the stress increased to more than 10 MPa and the strain was 0.7 ~ 1.0, the stress continued to grow upward, the stress‒strain relationship was positive, and the curve was steeper, indicating that the strength increased faster. The J3 curve fluctuated, and the test block showed crack expansion. The curve reached the ultimate load, the stress no longer increased, the specimen cracks expanded to penetration damage, the curve fluctuated down, and the bearing capacity decreased. The curve was divided into two parts. One part was in the rising stage. Starting from the 0 point, microcracks appeared during the rising stage of the curve, and with increasing load, the cracks expanded. When the ultimate stress was reached, the cracks were still expanding, although the stress no longer increased, and the curve went downward. Microcracks appeared during the rising stage with limited crack expansion. In the falling stage, the falling curves of J2 and J3 under the stress‒strain curve were more turbulent, the stress fluctuated and decreased, there was obvious residual strength, and the crack expansion increased.

### Strength characteristics of the assembly specimens

**Figure 5:**
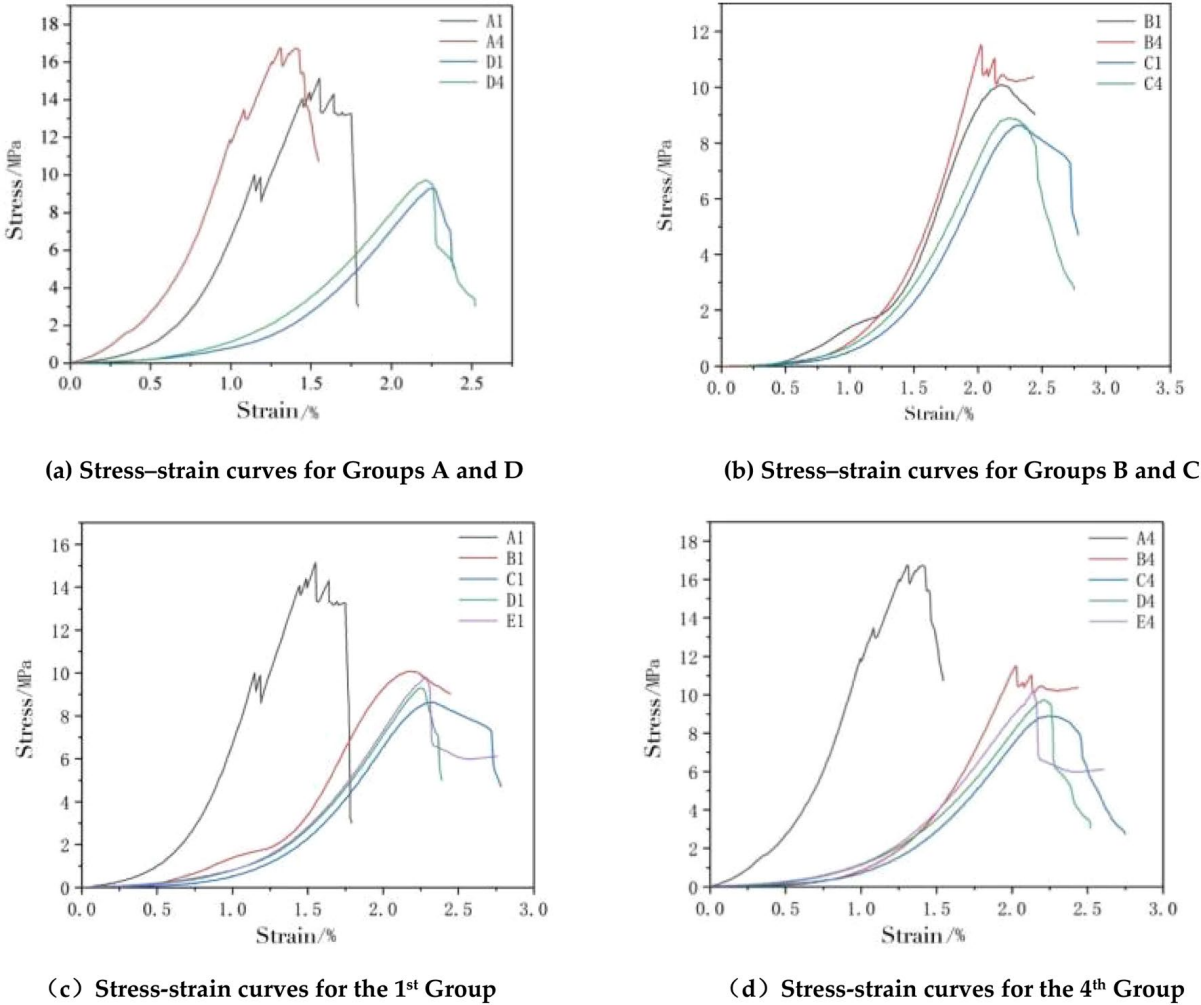
Stress–strain curves of the composite body.

The combination specimens were first compared with Group A and Group D. For the two specimens of Group A in Fig. 5a, the compressive strength of the specimens was higher than that of the two specimens of Group D regardless of whether the CPB was compressed on the upper side or the net slurry specimen was on the upper side. The curve trends of the two groups are different, and the curve trends of Group D were roughly the same as those of the CPB. Before the curve stress reached 4 MPa, both groups had concave bending curves. The difference was that the curve of Group D rose slowly and the slope was lower. When the stress of Group A continued to increase, there were fluctuations, indicating that cracks appeared at this moment. When the sustained stress was loaded to the peak, two wave peaks appeared in Group A. After reaching the peak stress, the curve began to show a downward trend.

As shown in Fig. 5b, when Group B was compared with Group C, the overall strength of Group B was higher than that of Group C, and the cut-line modulus presented was also higher than that of Group C, which was consistent with the compressive strength relationship of the assemblage. Figure 5c shows the stress‒strain diagram of Group 1 for each height ratio of the assemblage. A1 had the highest strength, as shown in Fig. 5d for the stress‒strain diagram of Group 4 for each height ratio of the assemblage, and the overall strength of Group 4 was higher than that of Group 1.

Table 3 presents the mean peak height and mean secant modulus for different height ratios, with the CPB part on the upper side and the surrounding rock part on the upper side.

**Table 3:** Assembly test sheet.

Specimen number	Average peak intensity/MPa	Average secant modulus/GPa
A1 ~ A3	15.15	7.25
A4 ~ A6	16.74	9.72
B1 ~ B3	11.51	3.69
B4 ~ B6	11.93	4.68
C1 ~ A3	8.63	2.72
C4 ~ A6	8.89	2.86
D1 ~ D3	9.3	2.91
D4 ~ D6	9.73	3.30
E1 ~ E3	10.08	3.49
E4 ~ E6	10.87	3.84

The above table shows the results of this type of compression test. From 1 to 3, the compressive strength and cut line modulus of the specimen from Group A were the highest, 15.15 MPa and 7.25 GPa, respectively. The results for Group B were the second highest, with compressive strength decreased by 24% and cut line modulus decreased by 49%. For Group C, the compressive strength decreased by 43%, and the cut line modulus decreased by 62%. The compressive strength of Group D was reduced by 39% and the modulus of cut line was reduced by 60%. The compressive strength of Group E was reduced by 33% and the modulus of cut line was reduced by 57%. From Groups 1 to 3, the order of compressive strength was A-B-E-D-C, and the order of cut line modulus was A-B-E-D-C.

From Groups 4 to 6, the compressive strength and cut line modulus of specimen A were the highest, 16.74 MPa and 9.72 GPa, respectively. The results for Group B were the second highest, with a 29% reduction in compressive strength and a 52% reduction in cut line modulus. Group C had the highest compressive strength and cutline modulus, respectively. The compressive strength of Group C decreased by 47%, and the cut line modulus decreased by 71%. The compressive strength of Group D decreased by 42%, and the cut line modulus decreased by 66%. The compressive strength of Group E decreased by 35%, and the cut line modulus decreased by 60%. From Groups 4 to 6, the order of compressive strength from largest to smallest was A-B-E-D-C, and the order of cut line modulus from largest to smallest was A-B-E-D-C.

**Figure 6:**
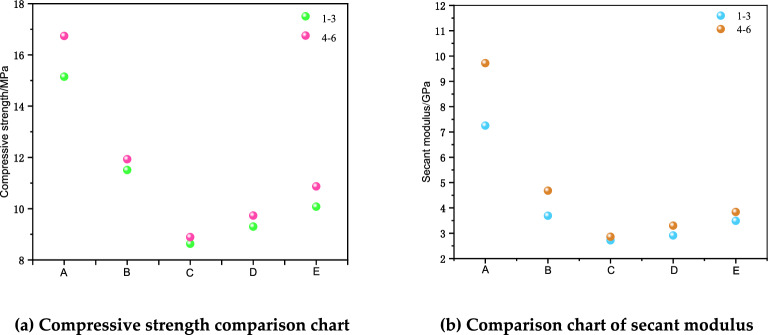
Diagrams of compressive strength and secant modulus.

For the two types, Groups 1 ~ 3 and Groups 4 ~ 6, for the specimens with the same height ratio, Groups 4 ~ 6 were higher than Groups 1 ~ 3 in both compressive strength and cut-line modulus. When the height ratio was Group A, the compressive strength and cut line modulus of the assemblage were most obviously affected by the different distributions above and below the surrounding rock-CPB. From the results, the compressive strength and cut line modulus of the two groups were slightly different, but their values were similar. It was obvious from the above test data that the mechanical parameters, such as compressive strength and cut line modulus, of different composite specimens were not the same under the influence of different height ratios and different pressures on the upper side of the material. To analyze the specific influence of different height ratios and different pressures on the compressive strength and cut line modulus of the structural specimens, compressive strength, cut line modulus and cut line modulus curves were obtained for different heights and different pressures. The relationship curves of compressive strength and cut line modulus with different heights and different compression conditions are shown in Fig. 6.

The analysis shows that the peak strength of the surrounding rock was 4 ~ 5 times that of the CPB specimen, the peak strength of the combined specimen was 1–2 times that of the CPB specimen, and the peak strength of the surrounding rock specimen was 2 ~ 5 times that of the combined specimen. For the three specimens, the preliminary stress–strain slope of the CPB was smaller, the curve increase rate was smaller for the other two specimens, and the growth was slightly slower. The slope was steepest for the surrounding rock and second steepest for the combined body. For the peak strength and modulus of elasticity, the surrounding rock specimen were the largest, second-largest for the composite specimen, and smallest for the CPB specimen.

## Curve fits curves and functions

There was a relationship between the structural specimens of assemblies with different height ratios and compressive strengths. When the height of the surrounding rock increased beyond a certain limit, the rebound deformation of the surrounding rock was violent, which led to a certain fluctuating trend for the overall strength of the structural specimen. Therefore, we used various fitting methods to analyze the relationship between different height ratios of the assemblage and compressive strength.

**Figure 7:**
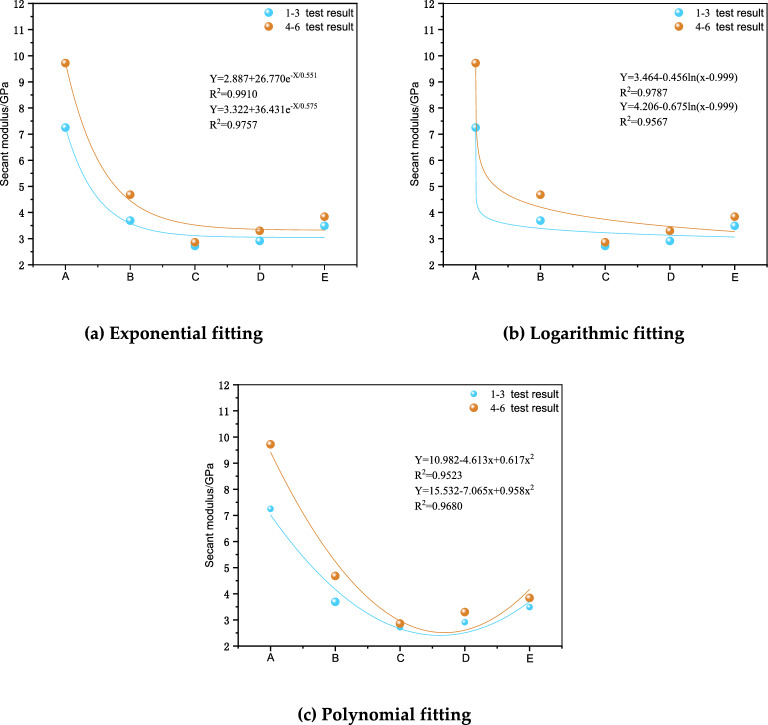
Secant modulus fitted curves.

Figure 7 shows the fitted cut line modulus curves and functional relationships for exponential, logarithmic and polynomial fits for each combination through different height ratios as variables. The results of the fits showed that the exponential, logarithmic and polynomial fits resulted in maximum R2 values of 99.10% for Groups 1 to 3 and 97.57% for Groups 4–6, which were all good fits. The exponential fitting method had the highest complex correlation coefficient, which indicated that the exponential fitting method could better reflect the quantitative relationship between different height ratios of the assemblage and the cut line modulus.

**Figure 8:**
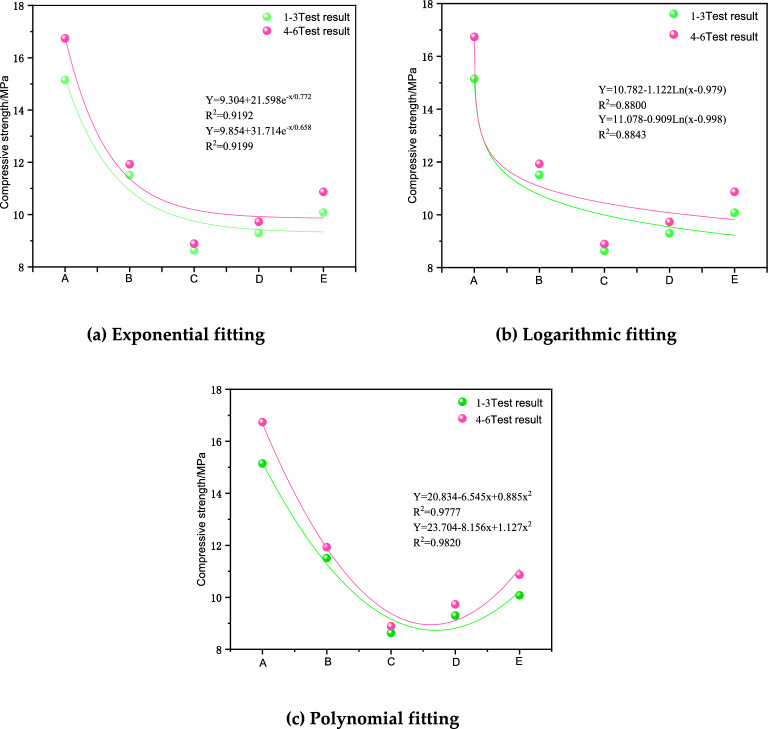
Strength fitting curves.

Figure 8 shows the fitted curves and functional relationships of compressive strength fitted through each combination with different height ratios as variables. The fitted results showed that a higher complex correlation coefficient indicated a better correlation between the height ratio and the compressive strength, with maximum R2 values of 97.77% for Groups 1–3 and 98.20% for Groups 4–6 after exponential, logarithmic and polynomial fitting. The largest complex correlation coefficients were found for the polynomial fitting method, indicating that the polynomial fitting method could better reflect the quantitative relationship between different height ratios and the compressive strength of the assemblies.

The cut line modulus, which reflects the average stiffness of the rock in rock mechanics, was applied to study the difference. Here, the slope of the line connecting the 50% peak compressive strength point with the origin of the coordinates was chosen as the value of the cut line modulus, i.e.,

1 $$ {\text{E}}_{{{\text{s50}}}} = \frac{{{\upsigma }_{{{\text{p50}}}} }}{{{\upvarepsilon }_{{{\text{p50}}}} }} $$

where: ${\text{E}}_{{{\text{s50}}}}$ is the cutline modulus; ${\upsigma }_{{{\text{p50}}}}$ is the 50% stress value of the uniaxial compressive strength; and ${\upvarepsilon }_{{{\text{p50}}}}$ is the corresponding axial strain value of the specimen.

The complex correlation coefficients of the three fitting methods in Groups 1 ~ 3 were 0.9192, 0.8800, and 0.9777. The complex correlation coefficients of the exponential and logarithmic and polynomial fitting in Groups 4 ~ 6 were 0.9199, 0.8843, and 0.9843, respectively. All of these showed better fitting characteristics, and the R2 values were larger for Groups 4–6. The highest results for polynomial fitting indicated that the polynomial fitting could better characterize the quantitative relationship between different height ratios and the compressive strength of the cemented fill. In addition, the interaction between the surrounding rock and the CPB affected the compressive strength of the composite specimen. The overall strength of the composite body was significantly weakened with the increasing thickness of the upper material, and the cut line modulus and strength were reduced. When the height ratio reached 3:1, the height reached a certain limit, and the strength showed rising fluctuations. Compared with the specimen of Group C with a height ratio of 3:1, the stress curve of the specimen of Group D appears to rebound, and there was a certain rise in the elastic modulus. However, due to the low overall strength, the damage started at the lower peak strength.

## Warping of the specimens

### Deformation characteristics of the CPB specimens

**Figure 9:**
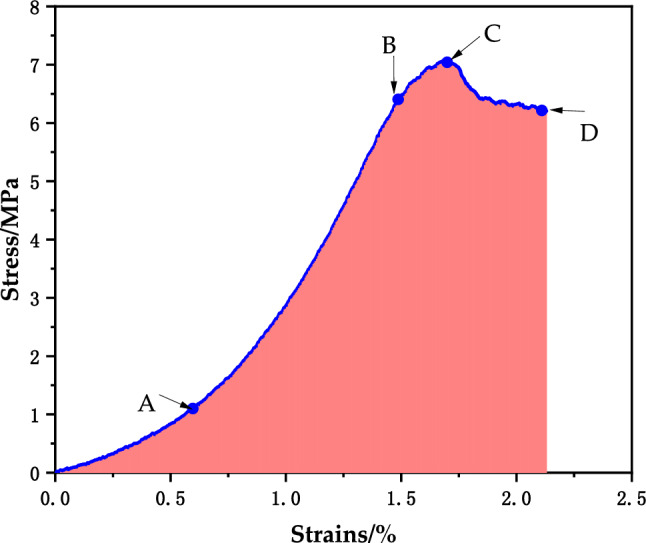
T3 stress‒strain curve.

The stress‒strain curve of the CPB is shown in Fig. 9 for T3 as an example. The first stage was the microfracture closure stage. As the load compressed the specimen and the load increased. The specimen first contacted the press and the pressure-bearing plate, and under the action of the load, the pores inside the CPB specimen were compressed at the beginning of the loading, and the stress‒strain curve was depressed downward. The curve showed an upward trend, and with the growth of strain, the stress also grew. The second stage was the elastic stage, in which the stress and strain increased positively, the curve rose faster, the curve was steeper, close to a straight line, and the slope of the line could be regarded as the elastic modulus. With increasing load, the specimen was compressed and compacted. The third stage was the yielding stage, and with the continuous increase in load, the crack expanded steadily. The stress and strain curves were no longer proportional, the pores continued to be compacted, and further expansion of the end cracks occurred at this stage. The cracks showed unstable expansion, and the curve showed an upward convex shape. The stress of the specimen gradually reached the maximum point, the slope decreased to zero, its value was the uniaxial compressive strength, and the CPB specimen underwent cracking and started to be destroyed. The fourth stage was the damage stage. The crack of the specimen has been produced, and then the load continues to be applied. The crack expansion intensified, which led to rapid expansion of the crack through the destruction of the CPB. The curve showed a downward trend, and the slope was negative, slowly decreasing. For ductile damage, there was a large residual stress.

### Deformation characteristics of the net pulp specimens

**Figure 10:**
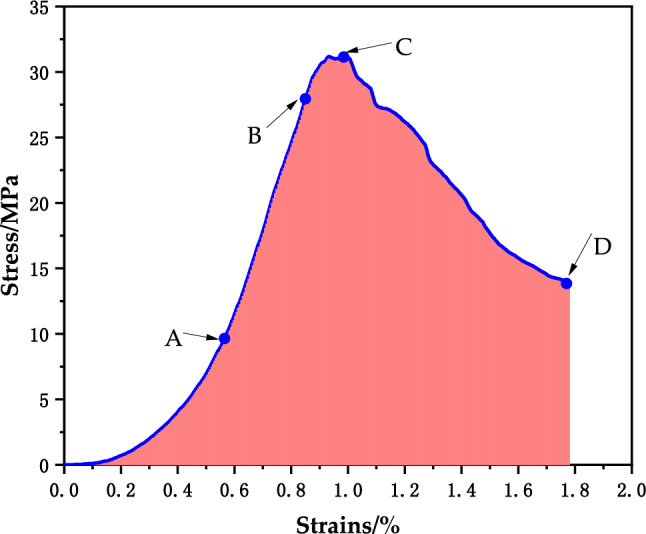
J1 stress‒strain curve.

The stress‒strain diagram of the surrounding rock specimen is shown in Fig. 10. Taking specimen J1 as an example, the curve was divided into four stages. The first stage was the microfracture closure stage, the surrounding rock specimen was in the loading stage, the specimen was compacted downward, the internal pores were compressed and compacted, and the curve was depressed downward. The second stage was the elastic stage, the curve in this stage was close to a straight line, with the most rapid growth, and the slope was almost constant and could be used as the elastic modulus. The third stage was the yielding stage, the crack began to expand, the slope of the curve decreased, and plastic deformation occurred. With continued loading, the crack underwent unstable expansion, and the slope decreased to zero. The fourth stage was the damage stage, and the curve after the peak exhibited a decreasing trend and fluctuating shape with obvious residual strength after the peak. The sandstone mainly showed ductile damage.

### Deformation characteristics of the assembly specimen

**Figure 11:**
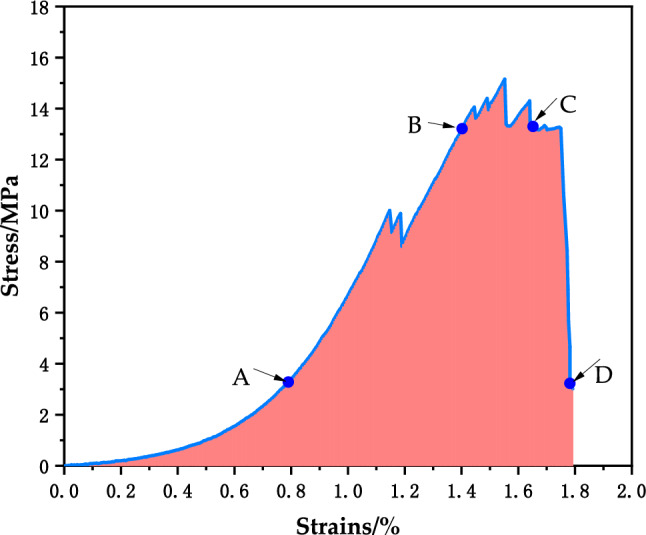
A1 stress‒strain curve.

The stress‒strain curve of the CPB-sandstone composite specimen is shown in Fig. 11 as an example. The first stage was the compression-density stage, the composite was in the early stage of loading, and the curve was slightly concave. The second stage was the elastic stage, the curve in this stage showed nearly linear growth, the slope was almost constant, and the slope could be used as the elastic modulus of the specimen. The third stage was the crack stable expansion stage, and the slope of the curve was still almost unchanged, but the combination began to undergo plastic deformation. Continuing to load the crack caused the unstable expansion stage, and the curve was convex upward until the slope of the curve gradually reduced to zero. In the fourth stage for the peak after the destruction stage, the curve section of the stress showed a rapid decrease, the peak after the residual strength was not obvious, and the main performance of the combination was brittle damage.

### Comparative analysis of deformation characteristics of filling, sandstone, and composite specimens

In the first stage, the surrounding rock, CPB and composite specimens all exhibited an obvious depression. In the second stage, the CPB, sandstone and composite specimens all showed nearly linear growth, but the growth was more obvious for the composite specimen. In the third stage, the slope of the curve of each specimen gradually decreased to zero. In the fourth stage, the CPB specimen maintained a high residual stress, the stress fluctuated and decreased for the sandstone specimen, and the stress fluctuated and decreased for the composite specimen. In the fourth stage, the CPB specimens maintained high residual stress, the sandstone specimens underwent fluctuating decreasing stress and the assemblage specimens underwent rapidly decreasing stress.

The bending deformation damage characteristics of cast and natural stacked assemblages were different, and the strengths were also different. When the rock specimen was subjected to axial pressure only and there was no lateral pressure, the damage form was mainly tensile damage and shear damage where the shear damage form was divided into conjugate bevel and single bevel shear damages. The test CPB, sandstone and composite specimens underwent tensile damage and single bevel shear damage, and a variety of damage forms coexisted. The rock specimens were damaged, small pieces spalled and made brittle cracking sounds, and there were fluctuations in the decline and residual strength, which corresponded to ductile damage. The CPB damage was ductile without a loud noise, and the combined specimen underwent a change to brittle damage with a rapid drop in stress.

## Conclusions

1. The surrounding rock specimen was the strongest, followed by the combined body, and the CPB was the weakest. The average values of the compressive strengths of the CPB and surrounding rock were 7.13 MPa and 31.35 MPa, respectively, and the strength of the CPB under the upper layer of surrounding rock was higher than that of the upper layer of the CPB combination. From Groups 1 ~ 3 and 4 ~ 6, the order of compressive strengths from large to small was A-B-E-D-C, and the order of cut line moduli from large to small was A-B-E-D-C.
2. From the stress‒strain diagram, the damage process of CPB specimens under uniaxial compression was divided into four stages: microfracture closure stage, elastic stage, yielding stage and damage stage. The CPB specimens fell gently in the damage stage, the surrounding rock specimens fluctuated and fell in the damage stage, and the combined body specimens fell rapidly.
3. The CPB and surrounding rock specimens had obvious post-peak residual strength after the failure stage, which was mainly manifested as ductile failure. The combination specimen was directly destroyed after the elastic stage, and the bearing capacity was instantly lost, which eventually led to the instability and failure of the entire combination sample, which was brittle failure.

According to research published in the literature, most materials, such as coal-rock and cement tailings, were selected in previous research on the mechanical properties of the assemblage to study the mechanical properties and the mechanism of synergistic deformation, while research on RHA slag was less common. In this paper, for the first time, the use of RHA and slag as cementitious materials, the use of alkali excitation to stimulate the activity, and the use of tailings sand as aggregate were explored to prepare a new type of CPB. Through the uniaxial compression test, the mechanical properties and damage characteristics of the three kinds of specimens were studied, and an in-depth analysis of the change stage of its specimens was conducted. Results for different types of layering and different mixing ratios showed that interactions between the surrounding rock and CPB affected the compressive strength of the assemblage specimens.

In the experimental process, uniaxial compression experiments were used to study the mechanical strength characteristics based on the compressive strength and cut line modulus. The results were fitted with functions to analyze the relationship between the height ratio of the assemblage and the compressive strength. Additionally, the deformation characteristics of different specimens were analyzed through their stress‒strain curves. In future research, true triaxial tests will be used to study the mechanical properties of filling bodies as well as the acoustic characteristics and damage forms of assemblies with different thicknesses. These future studies will make it possible to continue to explore this subject at a deeper level.

## Data Availability

Data can be obtained from the corresponding authors upon reasonable request.

## References

[1] Singh KB, Dhar BB (1997). Sinkhole subsidence due to mining. Geotech. Geol. Eng..

[2] Liu Z, Mei G, Sun Y, Xu N (2021). Investigating mining-induced surface subsidence and potential damages based on SBAS-InSAR monitoring and GIS techniques: A case study. Environ. Earth Sci..

[3] Lian X (2020). Law of movement of discontinuous deformation of strata and ground with a thick loess layer and thin bedrock in long wall mining. Appl. Sci..

[4] Guo J-X, Ding K, Jian Y (2019). Study on compaction characteristics of paste filling and its application. Geotechn. Geol. Eng..

[5] Jiang N, Wang C, Pan H, Yin D, Ma J (2020). Modeling study on the influence of the strip filling mining sequence on mining-induced failure. Energy Sci. Eng..

[6] Pacewska B, Wilińska I (2020). Usage of supplementary cementitious materials: advantages and limitations: Part I. C-S–H, C–A–S–H and other products formed in different binding mixtures. J. Therm. Anal. Calorim..

[7] Cao H, Gao Q, Zhang X, Guo B (2022). Research progress and development direction of filling cementing materials for filling mining in iron mines of China. Gels.

[8] Nicoara AI (2020). End-of-life materials used as supplementary cementitious materials in the concrete industry. Materials.

[9] Mounika G, Baskar R, Rama SKJ (2022). Rice husk ash as a potential supplementary cementitious material in concrete solution towards sustainable construction. Innov. Infrastruct. Solut..

[10] Anwar M, Miyagawa T, Gaweesh M (2000). Using rice husk ash as a cement replacement material in concrete. Waste Manag. Ser..

[11] Jw A (2021). Action mechanism of rice husk ash and the effect on main performances of cement-based materials: A review. Construct. Build. Mater..

[12] Abd-Ali MS, Kadhim SJ (2020). Experimental study on influence of Iraqi rice husk ash as supplementary material on the performance of concrete. IOP Conf. Ser. Mater. Sci. Eng..

[13] Adesina PA, Olutoge FA (2019). Structural properties of sustainable concrete developed using rice husk ash and hydrated lime. J. Build. Eng..

[14] Madandoust R, Ranjbar MM, Moghadam HA, Mousavi SY (2011). Mechanical properties and durability assessment of rice husk ash concrete. Biosyst. Eng..

[15] He Z-H, Li L-Y, Du S-G (2017). Creep analysis of concrete containing rice husk ash. Cem. Concr. Compos..

[16] Chouhan P, Jamle S, Verma M (2017). Effect of silica fume on strength parameters of concrete as a partial substitution of cement. IJSART3.

[17] Peng Y, Hu S, Ding Q (2009). Dense packing properties of mineral admixtures in cementitious material. Particuology.

[18] Zhang T, Ma B, Jiang D, Jiang Q, Jin Z (2021). Comparative research on the effect of various mineral admixtures on the early hydration process of cement. Construct. Build. Mater..

[19] Wang J, Fu J, Song W, Zhang Y (2021). Mechanical properties, damage evolution, and constitutive model of rock-encased backfill under uniaxial compression. Construct. Build. Mater..

[20] Fu J, Zhang B, Tan Y, Wang J, Song W (2023). Study on creep characteristics and damage evolution of surrounding rock and filling body (SR-FB) composite specimens. J. Mater. Res. Technol..

[21] Li F, Yin D, Wang F, Jiang N, Li X (2022). Effects of combination mode on mechanical properties of bi-material samples consisting of rock and coal. J. Mater. Res. Technol..

[22] Zhao K (2022). Strain-rate effects on the crack evolution pattern and damage characteristics of cemented paste backfill. Geotech. Geol. Eng..

[23] He Z, Zhao K, Yan Y, Ning F, Song Y (2021). Mechanical response and acoustic emission characteristics of cement paste backfill and rock combination. Construct. Build. Mater..

[24] Liu X, Tan Y, Ning J, Lu Y, Gu Q (2018). Mechanical properties and damage constitutive model of coal in coal-rock combined body. Int. J. Rock Mech. Min. Sci..

[25] Pan B, Yu W, Shen W (2021). Experimental study on energy evolution and failure characteristics of rock–coal–rock combination with different height ratios. Geotech. Geol. Eng..

[26] Chen Y, Zuo J, Liu D, Li Y, Wang Z (2021). Experimental and numerical study of coal-rock bimaterial composite bodies under triaxial compression. Int. J. Coal Sci. Technol..

[27] Yu X, Kemeny J, Tan Y, Song W, Huang K (2021). Mechanical properties and fracturing of rock-backfill composite specimens under triaxial compression. Construct. Build. Mater..

[28] Li Z (2022). Deformation and failure characteristics of bimaterial samples consisting of sandstone and cemented coal gangue-fly ash backfill under uniaxial loading. Minerals.

[29] Tan Y-Y (2020). The mechanical and microstructural properties of composite structures made of a cement-tailing backfill and rock core. Minerals.

[30] Liang W (2022). Experimental study on the interaction between backfill and surrounding rock in the overhand cut-and-fill method. Minerals.

[31] Li G (2023). The creep behavior of rock shear seepage under different seepage-water pressures. Mech. Time-Depend. Mater..

[32] Zhao W, Ji C, Sun Q, Gu Q (2022). Preparation and microstructure of alkali-activated rice husk ash-granulated blast furnace slag tailing composite cemented paste backfill. Materials.

